# Vision-Related Quality of Life in Patients with Inactive HLA-B27–Associated-Spectrum Anterior Uveitis

**DOI:** 10.1371/journal.pone.0146956

**Published:** 2016-01-25

**Authors:** Lisette Hoeksema, Leonoor I. Los

**Affiliations:** 1 Department of Ophthalmology, University Medical Center Groningen, University of Groningen, Groningen, the Netherlands; 2 W.J. Kolff Institute, Graduate School of Medical Sciences, University of Groningen, Groningen, the Netherlands; Boston University School of Medicine, UNITED STATES

## Abstract

We investigated the vision-related quality of life (VR-QOL) in patients with HLA-B27 associated anterior uveitis (AU). The study was conducted in 2012 at the ophthalmology department of the University Medical Center of Groningen. We included AU patients who were HLA-B27 positive and/or were diagnosed by a rheumatologist with an HLA-B27 associated systemic disease. Sixty-one of 123 (50%) adult patients participated. All patients filled-out the National Eye Institute Visual Functioning Questionnaire-25 (NEI VFQ-25), Beck Depression Inventory (BDI-II), social support lists and an additional questionnaire for gathering general information. Medical records were reviewed for clinical characteristics. Analyses were conducted on various patient and ocular characteristics. We compared our NEI VFQ-25 scores with those previously found in the literature. Our main outcome measures were VR-QOL scores and their associations with various general patient and ocular characteristics. We found that the NEI VFQ-25 mean overall composite score was 88.9±8.8, which is relatively high, but lower than that found in a normal working population. The mean general health score was 47.4±20.8, which is lower than in patients with other ocular diseases. Patients with a systemic disease scored significantly lower on general health and VR-QOL, compared to patients without a systemic disease. Patients with a depression (6/59 (10%)) frequently had ankylosing spondylitis (5/6 patients) and they scored significantly worse on VR-QOL. We concluded that patients with HLA-B27 associated AU have a relatively high VR-QOL. However, the presence of a systemic disease is associated with lower VR-QOL and general health scores. In addition, depression is associated with a lower VR-QOL.

## Introduction

Anterior uveitis (AU) is the most common form of uveitis and it is commonly associated with HLA-B27 associated diseases, such as ankylosing spondilitis, Crohn’s disease, reactive arthritis and psoriasis. Also, HLA-B27 positivity without apparent systemic disease may be associated with AU.[1] This type of uveitis is often recurrent and it can occur uni- or bilaterally. Complications like high intraocular pressure, glaucoma, cataract, posterior synechiae and dry eyes are seen in HLA-B27 associated AU.[2] The visual acuity (VA) can decrease temporarily or permanently because of recurrent inflammation and complications of AU. All these characteristics can affect a patient’s quality of life.

Previous studies indicated a poorer visual functioning and a lower general health status in uveitis patients compared to healthy subjects.[3] Most studies evaluated VR-QOL in heterogenous groups of uveitis patients.[3–5] Qian et al. found an inverse correlation between National Eye Institute Visual Function Questionnaire-25 (NEI VFQ-25) scores and best-corrected visual acuity (BCVA) and between NEI VFQ-25 and Beck Depression Inventory II (BDI-II) scores in patients with non-infectious ocular inflammatory disease.[4] Schiffman et al. reported that non-infectious uveitis patients with more severe uveitis have poorer visual functioning and general health status than patients with milder disease.[3] A few studies looked at VR-QOL in specific uveitis patient groups. Kuiper et al. observed that VR-QOL is impaired in patients with birdshot chorioretinopathy and that VR-QOL composite scores were related to BCVA, but not related to age or duration of uveitis.[6] In a previous study, we found that VR-QOL is only mildly reduced in herpetic AU.[7] These findings suggest differences in impact on VR-QOL in various uveitis entities. For proper patient counseling, it would therefore be useful to examine VR-QOL in the different uveitis entities separately and to investigate the ocular and patient characteristics that may influence VR-QOL.

The purpose of the present study is to evaluate the VR-QOL in a group of patients with an HLA-B27 associated AU. We hope to identify clinical features that are associated with a lower quality of life in this patient group. Identifying such features may help to develop targeted screening and intervention measures in the future.

## Methods

The Medical Ethical Committee of the University Medical Center of Groningen ruled that approval was not required for this study, since the study is not a medical scientific study with people as defined in the *Medical Research Involving Human Subjects Act*. The study was conducted according to the tenets of the Declaration of Helsinki.

### Patients

We selected all patients from an existing database, containing uveitis patients who are currently being treated or who have been treated for uveitis at the ophthalmology department of the University Medical Center Groningen, which is a tertiary referral center.

We identified 123 adult (18 years or older) patients with HLA-B27 associated AU. Patients were HLA-B27 positive and/or were clinically diagnosed with an HLA-B27 associated systemic disease by a rheumatologist. Patients with ankylosing spondylitis were diagnosed following the Assessment of SpondyloArthritis international Society classification criteria (ASAS criteria). Therefore patients with sacroiliitis on imaging and ≥ one axial spondyloarthritis criteria, have not always been tested for HLA-B27 positivity. [8] Patients with other forms or possible causes of uveitis were excluded.

### Data

All suitable 123 patients received the following questionnaires by mail: the National Eye Institute Visual Functioning Questionnaire-25 (NEI VFQ-25), the Beck Depression Inventory (BDI-II), Social Support List–Interactions (SSL-I), Social Support List–Discrepancies (SSL-D) and an additional questionnaire for gathering general information. They also received an information letter and an informed consent form. Patients could complete the questionnaires and sign the informed consent form at home and return them by mail. We contacted all patients by phone, to ask if they needed assistance in filling out the questionnaire. All patients were literate.

In this study, we used the Dutch version of the NEI VFQ-25. This validated [9,10] questionnaire measures the VR-QOL and has been developed by the National Eye Institute. This self-administered questionnaire consists of a base set of 25 vision-targeted questions representing 11 vision-related subscales, plus an additional single-item general health rating question. The overall composite score (OCS) is the average of the vision-targeted subscale scores, without the general health score. In this questionnaire, a score of 0 corresponds to the lowest and of 100 to the highest VR-QOL. There are 12 subscales, each consisting of one or more questions. These subscales are general health, general vision, ocular pain, near activities, distance activities, vision specific social functioning, vision specific mental health, vision specific role difficulties, vision specific dependency, driving, color vision and peripheral vision.

The BDI-II is a validated [11] self-administered questionnaire evaluating how a patient feels and experiences things. It consists of 21 questions and each question can be answered on a four-point scale ranging from 0 to 3. All scores are added to provide a total score, with a maximum of 36. The total score gives an estimation of the severity of an existing depression. A total score of 0 to 13 corresponds with no depression, of 14 to 19 with a mild depression, of 20 to 28 with a moderately severe depression and of 29 to 63 with a severe depression.

The SSL-I and SSL-D are questionnaires developed and validated by the University of Groningen. They each consist of 34 four-choice questions, resulting in scores ranging from 1–4. The SSL-I questionnaire measures the social interactions between patients and persons with whom they interact. The maximum score is 136, a high SSL-I score corresponds with sufficient social support. The SSL-D questionnaire measures if the received social support corresponds with the desired social support. The maximum score is 102, a high SSL-D score corresponds with a deficiency in desired social support.

The additional questionnaire gave us the following information: whether the uveitis was active at the time of completing the questionnaire, medical history (including chronic diseases or diseases with a large impact, recently or in the past), medication use (ocular and other medication) and history of depression and the need for treatment.

The following information was collected by examining medical records: current age, sex, uni- or bilateral AU, presence of a systemic disease, follow-up (time between the start of the first uveitis episode and the end of the last uveitis episode), total number of uveitis episodes, total time of active disease in months, remission time (time between the end of the last uveitis episode and the date on the questionnaire), Snellen VA, ocular complications (elevated intraocular pressure (IOP), glaucoma, cataract, secondary cataract, dry eyes and current treatment for dry eyes, cystoid macular edema (CME), papillitis, scleritis and posterior synechiae) and present activity of the uveitis. To evaluate a possible bias by inclusion, the following data were collected from the patients who did not return the questionnaire: visual acuity at the end of follow-up, duration of follow-up, presence of systemic disease, bilaterality of disease (simultaneously bilateral or alternating between the right and left eye) and ocular complications.

Active uveitis was defined as ≥0.5+ cells in the anterior chamber.[12] Transiently elevated IOP was defined as a measured IOP > 20 mmHg without pressure reducing medication. Glaucoma was defined as the presence of visual field defects typical for glaucoma that were reproducible and could not be explained by other pathology, with or without glaucomatous disc abnormalities. Dry eyes were defined as the presence of dry eye symptoms and the need for artificial tears at any time.

### Statistics

Data were statistically analyzed using SPSS Statistics 20.0.0.1. The Mann-Whitney U test was used to compare continuous variables of two groups. The Kruskal–Wallis one-way analysis was used to compare continuous variables of more than two groups and the Mann-Whitney U test for post hoc analysis with a Bonferroni correction, using a critical value of 0.05 divided by the number of tests conducted. Correlations were assessed with the Spearman’s Rank Correlations test. For analyzing, Snellen VA was converted to the logarithm of the minimum angle of resolution (logMAR) equivalent. Statistical significance level was set at 0.05.

## Results

Sixty-one of 123 (50%) patients filled out the questionnaires and returned them by mail.

Table 1 gives an overview of the clinical characteristics of these patients. Male to female ratio was 3:2. Mean age was 55 ± 12 years. The group contained approximately the same percentage of patients with unilateral and bilateral disease. Since only a small minority (n = 2) of the patients had active AU at the time of completing the questionnaires, these were excluded from the analyses. Four (7%) patients indicated that they had had a depression in the past, diagnosed by a physician and medically treated. Forty-three (73%) patients were tested for HLA-B27 positivity and tested positive (100%), sixteen (27%) patients were not tested for HLA-B27, but were diagnosed with an HLA-B27 associated systemic disease by a rheumatologist. A systemic disease was present in 34 out of 59 (58%) patients and ankylosing spondylitis was the most common (44%) (Table 1). Ocular complications most frequently observed (in % of patients) were posterior synechiae (63%), elevated IOP (37%), dry eyes (29%), cataract (27%) and CME (14%). We checked and confirmed that all complications developed after the diagnosis of AU. The mean (± SD) of the NEI VFQ-25, BDI-ll, SSL-I and SSL-D scores are given in Table 1. The BDI-II scores showed that six patients had a mild depression at the time of filling out the questionnaire; five out of these six patients (83%) had ankylosing spondylitis.

**Table 1:** clinical characteristics of HLA-B27 associated AU patients and overall scores on questionnaires (N and (%) or Mean ± SD (range)).

	Patients who filled out the questionnaires	Patients who did not fill out the questionnaires	p
Number of patients	59	56	-
Female / male	23 (39%) / 36 (61%)	22 (39%) /34 (61%)	0.973
Age at completing questionnaire / study (yrs)	54 ± 12 (28–81)	46 ± 15 (19–90)	**0.001**
Unilateral / bilateral*	28 (47%) / 31 (53%)	21 (38%) / 35 (63%)	0.280
Total uveitis episodes	7.3 ± 6.5 (1–40)	6.8 ± 6.3 (1–24)	0.579
Time of active uveitis (months)	5.8 ± 5.0 (1–30)	5.5 ± 6.0 (1–30)	0.753
Follow-up time (yrs)	13.6 ± 9.7 (0.03–36.0)	9.9 ± 9.6 (0.02–29.8)	**0.039**
Remission time (yrs)	3.2 ± 2.8 (0.00–12.0)	3.4 ± 3.4 (0.09–15.4)	0.721
Depression in past^†^	4 (7%)	-	-
Present depression	6 (10%)	-	-
HLA-B27 tested (% of total group)	43 (73%)	44 (79%)	0.477
HLA-B27 positive (% of tested)	43 (100%)	44 (100%)	-
HLA-B27 not tested (% of total group)^‡^	16 (27%)	12 (21%)	0.477
Systemic disease	34 (58%)	27 (48%)	0.312
- Ankylosing spondylitis	26 (44%)	22 (39%)	-
- Reactive arthritis	3 (5%)	0 (0%)	-
- Crohn / Colitis ulcerosa	2 (3%)	2 (4%)	-
- Other	3 (5%)	3 (5%)	-
Ocular complications^§^	53 (90%)	46 (82%)	0.234
- Posterior synechiae	37 (63%)	29 (52%)	-
- Elevated IOP	22 (37%)	25 (45%)	-
- Dry Eyes^||^	17 (29%)	11 (20%)	-
- Cataract	16 (27%)	16 (29%)	-
- CME	8 (14%)	7 (13%)	-
- Secondary cataract	4 (7%)	2 (4%)	-
- Papillitis	3 (5%)	3 (5%)	-
- Glaucoma	2 (3%)	1 (2%)	-
NEI VFQ-25 OCS^#^	88.9 ± 8.8 (53.7–98.0)	-	-
BDI-II score	4.7 ± 5.3 (0–19)	-	-
SSL-I score	76.2 ± 15.3 (34–104)	-	-
SSL-D score	42.1 ± 11.5 (34–80)	-	-

AU: anterior uveitis, IOP: Intraocular Pressure, CME: Cystoid Macular Edema, OCS: Overall Composite Score, BDI: Beck Depression Inventory, SSL-I: Social Support List—Interactions, SSL-D: Social Support List—Discrepancies.

* Simultaneously bilateral or alternating between the right and left eye.

^†^ Diagnosed by a physician and medically treated.

^‡^ All patients that have not been tested for HLA-B27 positivity (n = 17), were diagnosed with an HLA-B27 associated systemic disease by a rheumatologist.

^§^ Developed during follow-up AU and in at least one eye.

^**||**^ Medication needed.

^#^ Average of vision-targeted subscale scores, without general health subscore.

Table 1 also gives an overview of the clinical characteristic of the patients who did not fill out the questionnaires, which was available in 56 out of 64 patients. Patients who filled-out the questionnaire, had a slightly longer follow-up (13,6±9,7 versus 9,9±9,6 years, p = 0,04) and were older (55±12 versus 46±15) as compared to patients who did not fill-out the questionnaires.

The mean (± SD) of the OCS and the subscales of the NEI VFQ-25 in relation to diverse patients’ characteristics and ocular variables are shown in Tables 2 and 3. The mean OCS in the total group was 88.9±8.8 and the mean general health score was 47.4±20.8.

**Table 2:** NEI VFQ-25 subscale scores and ocular composite score (OCS), Mean ± S.

		GH	GV	OP	NA	DA	VSSF	VSMH	VSRD	VSD	D	CV	PV	OCS*
		(n = 58)	(n = 58)	(n = 59)	(n = 59)	(n = 59)	(n = 59)	(n = 59)	(n = 59)	(n = 59)	(n = 54)	(n = 59)	(n = 59)	(n = 59)
Total group (n = 59)		47.4±20.8	78.3±10.8	73.1±20.3	88.0±15.2	90.8±12.3	97.2±8.7	88.7±9.2	86.4±17.4	97.5±8.9	85.3±14.2	99.6±3.3	94.1±12.6	88.9±8.8
*Sex*	- Male (n = 36)	46.5±23.3	76.7±10.1	71.9±21.6	88.0±14.3	91.2±12.6	97.2±9.5	88.7±9.9	86.8±17.7	97.0±10.6	88.9±12.1	99.3±4.2	95.1±11.7	88.9±9.4
	- Female (n = 23)	48.9±16.3	80.9±11.5	75.0±18.5	88.0±16.8	90.2±12.0	97.3±7.5	88.6±8.1	85.9±17.4	98.2±5.6	79.8±15.7	100.0±0.0	92.4±14.0	88.9±7.9
		*p = 0*.*582*	*p = 0*.*155*	*p = 0*.*618*	*p = 0*.*648*	*p = 0*.*618*	*p = 0*.*825*	*p = 0*.*720*	*p = 0*.*822*	*p = 0*.*906*	***p = 0*.*025***	*p = 0*.*424*	*p = 0*.*386*	*p = 0*.*675*
*Present age (yrs)*	- < 45 (n = 14)	58.9±21.0	82.9±10.7	70.5±21.1	96.4±7.1	94.6±7.0	99.1±3.3	87.9±6.7	90.2±16.4	100.0±0.0	87.5±10.2	100.0±0.0	98.2±6.7	91.6±4.6
	- 45–65 (n = 34)	43.9±19.8	77.6±10.9	72.1±20.7	84.6±17.1	89.7±13.6	96.0±11.0	87.9±9.8	85.7±17.2	96.6±10.9	84.4±14.7	99.3±4.3	92.6±14.5	87.6±9.7
	- > 65 (n = 11)	43.2±19.7	74.5±9.3	79.5±18.8	87.9±13.1	89.4±13.0	98.9±3.8	92.0±9.7	84.1±20.2	97.0±7.7	85.2±18.5	100.0±0.0	93.2±11.7	89.3±9.4
		*p = 0*.*060*	*p = 0*.*143*	*p = 0*.*504*	***p = 0*.*026***	*p = 0*.*546*	*p = 0*.*668*	*p = 0*.*288*	*p = 0*.*523*	*p = 0*.*244*	*p = 0*.*887*	*p = 0*.*692*	*p = 0*.*363*	*p = 0*.*588*
*Uveitis episodes*†	- 1 episode (n = 6)	50.0±22.4	86.7±10.3	75.0±20.9	88.9±10.1	98.6±3.4	100.0±0.0	90.6±9.5	85.4±20.0	100.0±0.0	85.0±9.1	100.0±0.0	100.0±0.0	91.9±5.1
	- > 1 episode (n = 52)	47.1±21.0	77.3±10.6	72.8±20.7	87.8±15.9	89.9±12.7	96.9±9.2	88.6±9.2	87.0±17.1	97.3±9.4	85.8±14.6	99.5±3.5	94.2±11.7	88.7±9.1
		*p = 0*.*734*	***p = 0*.*048***	*p = 0*.*866*	*p = 0*.*747*	*p = 0*.*059*	*p = 0*.*343*	*p = 0*.*618*	*p = 0*.*891*	*p = 0*.*343*	*p = 0*.*594*	*p = 0*.*734*	*p = 0*.*216*	*p = 0*.*499*
*Laterality*	- Unilateral (n = 28)	50.0±19.6	79.3±11.7	80.4±18.5	89.6±14.6	92.0±9.8	97.3±7.1	90.8±7.5	88.4±16.6	98.2±6.6	88.1±11.0	100.0±0.0	95.5±9.8	90.9±7.2
	- Bilateral (n = 31)	45.2±21.8	77.4±10.0	66.5±20.0	86.6±15.8	89.8±14.2	97.2±10.1	86.7±10.2	84.7±18.2	96.8±10.7	82.4±16.7	99.2±4.5	92.7±14.7	87.0±9.7
		*p = 0*.*208*	*p = 0*.*539*	***p = 0*.*009***	*p = 0*.*416*	*p = 0*.*877*	*p = 0*.*626*	*p = 0*.*096*	*p = 0*.*401*	*p = 0*.*211*	*p = 0*.*281*	*p = 0*.*342*	*p = 0*.*580*	*p = 0*.*052*
*Visual acuity*‡,§	- > 0.15 (n = 7)	46.4±9.4	68.6±10.7	75.0±22.8	77.4±20.8	77.4±20.8	91.1±18.7	81.3±15.3	69.6±23.8	88.1±20.9	81.9±23.2	96.4±9.4	85.7±19.7	79.5±16.1
	- ≤ 0.15 (n = 42)	47.0±21.1	78.5±10.4	71.7±20.3	89.7±12.6	92.5±9.4	98.2±5.9	89.6±7.8	88.1±15.1	98.4±5.6	86.9±13.1	100.0±0.0	94.6±11.8	89.9±6.4
		*p = 0*.*961*	***p = 0*.*023***	*p = 0*.*683*	*p = 0*.*079*	***p = 0*.*041***	*p = 0*.*146*	*p = 0*.*160*	***p = 0*.*027***	***p = 0*.*002***	*p = 0*.*880*	***p = 0*.*014***	*p = 0*.*140*	*p = 0*.*170*
*Systemic disease*	- No (n = 25)	61.5±18.0	80.8±9.3	79.5±21.6	92.0±10.1	95.0±7.2	99.5±2.5	91.5±7.8	89.5±14.3	99.7±1.7	89.1±12.7	100.0±0.0	98.0±6.9	91.9±5.5
	- Yes (n = 34)	37.5±16.6	76.5±11.5	68.4±18.3	85.0±17.6	87.7±14.2	95.6±11.0	86.6±9.6	84.2±19.3	95.8±11.5	82.5±14.8	99.3±4.3	91.2±14.9	86.6±10.0
		***p<0*.*001***	*p = 0*.*121*	***p = 0*.*045***	*p = 0*.*243*	***p = 0*.*044***	*p = 0*.*101*	***p = 0*.*039***	*p = 0*.*392*	*p = 0*.*063*	*p = 0*.*079*	*p = 0*.*391*	*p = 0*.*041*	***p = 0*.*027***

NEI VFQ-25: National Eye Institute Visual Functioning Questionnaire-25, GH: General Health, GV: General Vision, OP: Ocular Pain, NA: Near Activities, DA: Distance Activities, VSSF: Vision Specific Social Functioning, VSMH: Vision Specific Mental Health, VSRD: Vision Specific Role Difficulties, VSD: Vision Specific Dependency, D: Driving, CV: Color Vision, PV: Peripheral Vision, OCS: Overall Composite Score.

* Average of vision-targeted subscale scores, without GH.

^†^ Missing data in one patient.

^‡^ At least one eye with LogMAR visual acuity > 0.15. Measured with Snellen chart and converted to LogMAR visual acuity, measured within six months before or after completing the NEI VFQ-25.

^§^ Missing data in ten patients.

**Table 3:** NEI VFQ-25 subscale scores and ocular composite score (OCS), Mean ± SD.

		GH	GV	OP	NA	DA	VSSF	VSMH	VSRD	VSD	D	CV	PV	OCS*
		(n = 58)	(n = 58)	(n = 59)	(n = 59)	(n = 59)	(n = 59)	(n = 59)	(n = 59)	(n = 59)	(n = 54)	(n = 59)	(n = 59)	(n = 59)
*Dry eyes*	- No (n = 42)	47.0±22.9	78.1±11.5	69.6±21.1	87.3±15.6	89.7±12.6	96.1±10.1	87.2±9.8	83.9±18.6	96.6±10.4	84.9±13.4	99.4±3.9	92.9±13.8	87.6±9.2
	- Yes (n = 17)	48.4±14.3	78.8±8.9	81.6±16.0	89.7±14.3	93.6±11.2	100.0±0.0	92.3±6.5	92.6±12.5	99.5±2.0	86.5±16.4	100.0±0.0	97.1±8.3	92.1±6.6
		*p = 0*.*711*	*p = 0*.*802*	***p = 0*.*041***	*p = 0*.*477*	*p = 0*.*180*	*p = 0*.*076*	*p = 0*.*064*	*p = 0*.*114*	*p = 0*.*260*	*p = 0*.*495*	*p = 0*.*525*	*p = 0*.*282*	***p = 0*.*049***
*Elevated IOP*	- No (n = 37)	49.3±21.6	78.4±8.7	74.0±20.9	89.0±12.0	91.7±10.4	98.3±6.0	90.5±7.7	89.2±14.8	98.9±4.5	85.4±11.0	100.0±0.0	96.0±9.3	90.1±6.6
	- Yes (n = 22)	44.0±19.2	78.1±14.0	71.6±19.7	86.4±19.7	89.4±15.0	95.5±11.9	85.5±10.6	81.8±20.7	95.1±13.3	85.3±18.4	98.9±5.3	91.0±16.4	86.8±11.4
		*p = 0*.*432*	*p = 0*.*848*	*p = 0*.*780*	*p = 0*.*645*	*p = 0*.*953*	*p = 0*.*252*	*p = 0*.*061*	*p = 0*.*195*	*p = 0*.*111*	*p = 0*.*438*	*p = 0*.*195*	*p = 0*.*253*	*p = 0*.*573*
*CME*	- No (n = 51)	47.5±21.0	78.8±11.0	71.6±20.6	88.2±14.6	90.5±11.4	97.8±6.5	89.1±8.1	87.0±16.6	98.5±5.2	84.4±14.1	100.0±0.0	95.1±10.0	89.3±7.4
	- Yes (n = 8)	46.9±20.9	75.0±9.3	82.8±16.3	86.5±19.4	92.7±17.5	93.8±17.7	85.9±14.8	82.8±23.1	90.6±20.1	93.1±13.4	96.9±8.8	87.5±23.1	86.4±15.4
		*p = 0*.*912*	*p = 0*.*367*	*p = 0*.*145*	*p = 0*.*862*	*p = 0*.*170*	*p = 0*.*859*	*p = 0*.*838*	*p = 0*.*766*	***p = 0*.*034***	*p = 0*.*092*	***p = 0*.*012***	*p = 0*.*506*	*p = 0*.*603*
*Posterior synechiae*†	- No (n = 21)	53.6±19.8	80.0±11.0	73.8±16.3	89.3±17.7	91.7±12.6	97.6±7.5	89.6±8.0	87.5±17.7	98.0±5.8	84.9±15.5	100.0±0.0	92.9±14.0	89.6±8.1
	- Yes (n = 37)	45.1±19.7	77.8±10.5	73.0±22.7	87.6±13.8	90.8±12.0	97.3±9.4	88.5±9.7	86.8±16.7	97.7±9.8	85.9±13.6	99.3±4.1	95.3±11.5	88.9±9.0
		*p = 0*.*123*	*p = 0*.*450*	*p = 0*.*993*	*p = 0*.*163*	*p = 0*.*493*	*p = 0*.*903*	*p = 0*.*740*	*p = 0*.*657*	*p = 0*.*699*	*p = 0*.*903*	*p = 0*.*451*	*p = 0*.*476*	*p = 0*.*827*
*Current depression*	- No (n = 53)	49.0±20.4	79.2 ±10.4	74.3±20.0	89.8±14.4	92.3±11.2	98.1±7.9	89.2±9.0	87.5±17.3	97.8±8.8	86.6±13.8	99.5±3.4	94.8±12.4	89.8±8.3
	- Yes (n = 6)	33.3±20.4	70.0±11.0	62.5±22.4	72.2±13.6	77.8±14.6	89.6±12.3	84.4±10.3	77.1±16.6	94.4±10.1	73.3±13.7	100.0±0.0	87.5±13.7	80.9±9.2
		*p = 0*.*106*	***p = 0*.*044***	*p = 0*.*167*	***p = 0*.*004***	***p = 0*.*012***	***P0*.*003***	*p = 0*.*212*	*p = 0*.*080*	*p = 0*.*134*	***p = 0*.*046***	*p = 0*.*737*	*p = 0*.*076*	***p = 0*.*022***
*Treatment uveitis*†,‡	- No (n = 49)	49.0±21.0	78.8±10.3	73.2±21.0	88.6±14.1	92.2±10.5	98.2±6.3	89.9±7.8	89.3±14.9	99.0±4.0	86.6±12.8	100.0±0.0	95.9±9.3	90.0±6.9
	- Yes (n = 9)	40.6±18.6	75.0±14.1	69.4±15.5	83.3±20.8	82.4±17.9	91.7±16.5	80.6±11.9	69.4±21.8	88.9±19.5	78.1±19.9	97.2±8.3	83.3±21.7	81.7±14.1
		*p = 0*.*421*	*p = 0*.*338*	*p = 0*.*646*	*p = 0*.*551*	*p = 0*.*069*	***p = 0*.*039***	***p = 0*.*018***	***p = 0*.*005***	***p = 0*.*003***	*p = 0*.*260*	***p = 0*.*020***	***p = 0*.*033***	*p = 0*.*075*
*Treatment systemic disease*§	- No (n = 15)	40.0±12.7	74.7±11.9	70.0±18.8	87.2±14.7	87.8±14.4	97.5±7.0	88.3±8.5	89.2±17.6	97.2±6.8	83.9±15.6	100.0±0.0	93.3±11.4	88.1±9.1
	- Yes (n = 19)	35.5±19.2	77.9±11.3	67.1±18.3	83.3±19.8	87.7±14.5	94.1±13.4	85.2±10.5	80.3±20.1	94.7±14.2	81.3±14.4	98.7±5.7	89.5±17.3	85.4±10.8
		*p = 0*.*451*	*p = 0*.*411*	*p = 0*.*710*	*p = 0*.*638*	*p = 1*.*000*	*p = 0*.*514*	*p = 0*.*394*	*p = 0*.*146*	*p = 0*.*883*	*p = 0*.*443*	*p = 0*.*374*	*p = 0*.*632*	*p = 0*.*395*

NEI VFQ-25: National Eye Institute Visual Functioning Questionnaire-25, GH: General Health, GV: General Vision, OP: Ocular Pain, NA: Near Activities, DA: Distance Activities, VSSF: Vision Specific Social Functioning, VSMH: Vision Specific Mental Health, VSRD: Vision Specific Role Difficulties, VSD: Vision Specific Dependency, D: Driving, CV: Color Vision, PV: Peripheral Vision, OCS: Overall Composite Score, IOP: Intraocular Pressure, CME: Cystoid Macular Edema.

* Average of vision-targeted subscale scores, without GH.

^†^ Missing data in one patient.

^‡^ Treatment of the uveitis and/or treatment of ophthalmic complications at the moment of completing the NEI VFQ-25.

^§^ Only patients with systemic disease (n = 35).

LogMAR VA higher than 0.15 (Snellen VA less than 7/10; a higher LogMAR VA corresponds to a lower Snellen VA), treatment of the uveitis at the time of completing the NEI VFQ-25, presence of a systemic disease and presence of a depression at the time of completing the NEI VFQ-25 were patients’ characteristics that were highly associated with a lower NEI VFQ-25 OCS and/or NEI VFQ-25 subscales. LogMAR VA higher than 0.15 in at least one eye, was correlated to significantly lower scores on general vision, distance activities, vision specific role difficulties, vision specific dependency and color vision. Treatment of the AU and treatment of ophthalmic complications at the moment of completing the NEI VFQ-25 were related to lower scores on the subscales, vision specific social functioning, vision specific mental health, vision specific role difficulties, vision specific dependency, color vision and peripheral vision. Patients with a systemic disease scored significantly lower on the OCS and on the subscales general health, ocular pain, distance activities, vision specific mental health and peripheral vision. Depression at the time of completing the NEI VFQ-25 was correlated to significantly worse scores on the OCS and on the subscales general vision, near activities, distance activities, vision specific social functioning and driving. These patients also scored lower on all other subscales, except color vision, but these subscales did not reach significance.

The patients’ characteristics that were moderately associated with lower NEI VFQ-25 OCS and/or NEI VFQ-25 subscales were older age, female gender, multiple uveitis episodes, bilateral AU, dry eyes and presence of CME. Younger patients (<45 years) scored significantly better (p = 0.026) on near activities compared to older patients (≥45 years). Post hoc analyses showed that near activity scores in patients <45 years versus those aged 45–65 years and patients <45 years versus those aged >65 years significantly differ (p = 0.008 and p = 0.049, respectively), whereas near activities scores did not significantly differ for patients between 45–65 years and patients >65 years (p = 0.714). Female patients scored significantly worse on driving. Patients who experienced only one uveitis episode had significantly higher general vision scores, compared with patients who experienced multiple uveitis episodes. Patients with bilateral AU scored significantly lower on ocular pain (indicating that they experienced more pain or discomfort around or in the eye). Patients with dry eyes scored significantly lower on ocular pain and on the OCS. Patients with CME scored lower on vision specific dependency and color vision. Elevated IOP, posterior synechiae and treatment of the systemic disease at the moment of completing the questionnaire showed no significant correlation with any of the NEI VFQ-25 outcomes.

The results of the Spearman’s Rank Correlations tests between NEI VFQ-25 subscale scores and OCS and of various patient and ocular characteristics are given in Table 4. Positive correlations were seen with age at completing the questionnaire and remission time. Age at completing the questionnaire was positively correlated to ocular pain and vision specific mental health. Remission time was positively correlated with general vision and vision specific mental health. Negative correlations were seen with higher LogMAR VA of the worst eye, follow-up time, BDI-II score and SSL-D score.

**Table 4:** Spearman’s Rank Correlations between studied variables and NEI VFQ-25 subscale scores and OCS.

	GH	GV	OP	NA	DA	VSSF	VSMH	VSRD	VSD	D	CV	PV	OCS*
Age at completing	-0.252	-0.242	**0.288**	-0.178	-0.154	0.065	**0.275**	-0.055	-0.143	-0.003	-0.116	-0.118	0.052
questionnaire	p = 0.056	p = 0.067	**p = 0.027**	p = 0.179	p = 0.243	p = 0.627	**p = 0.035**	p = 0.679	p = 0.280	p = 0.984	p = 0.383	p = 0.375	p = 0.697
LogMAR VA best eye	0.019	-0.258	**0.341**	-0.045	**-0.355**	-0.042	0.014	-0.011	**-0.290**	-0.054	-0.257	**-0.351**	-0.036
	p = 0.894	p = 0.073	**p = 0.016**	p = 0.757	**p = 0.011**	p = 0.775	p = 0.922	p = 0.940	**p = 0.041**	p = 0.719	p = 0.071	**p = 0.012**	p = 0.802
LogMAR VA worst eye	0.136	**-0.359**	0.262	-0.169	-0.248	-0.060	-0.023	-0.027	**-0.405**	-0.114	-0.247	-0.249	-0.102
	p = 0.358	**p = 0.012**	p = 0.069	p = 0.245	p = 0.086	p = 0.681	p = 0.877	p = 0.853	**p = 0.004**	p = 0.3452	p = 0.087	p = 0.085	p = 0.485
Total of uveitis episodes	-0.139	-0.139	-0.122	-0.103	-0.073	0.044	-0.034	0.008	-0.082	-0.053	-0.099	0.030	-0.091
	p = 0.303	p = 0.303	p = 0.362	p = 0.440	p = 0.588	p = 0.740	p = 0.798	p = 0.950	p = 0.539	p = 0.707	p = 0.458	p = 0.823	p = 0.495
Duration of active uveitis	-0.171	-0.160	-0.175	-0.246	-0.186	-0.155	0.035	-0.117	-0.125	-0.093	-0.025	-0.121	-0.177
	p = 0.207	p = 0.239	p = 0.192	p = 0.065	p = 0.166	p = 0.249	p = 0.794	p = 0.387	p = 0.353	p = 0.514	p = 0.856	p = 0.368	p = 0.189
Follow-up time	-0.170	**-0.341**	-0.145	-0.122	-0.076	-0.016	-0.055	0.031	**-0.277**	0.019	0.015	-0.125	-0.118
	p = 0.201	**p = 0.009**	p = 0.272	p = 0.357	p = 0.567	p = 0.905	p = 0.680	p = 0.816	**p = 0.034**	p = 0.891	p = 0.908	p = 0.346	p = 0.373
Remission time	0.123	**0.265**	0.136	0.121	0.067	0.177	**0.423**	0.141	0.181	-0.174	0.154	0.127	0.251
	p = 0.358	**p = 0.044**	p = 0.305	p = 0.362	p = 0.613	p = 0.180	**p = 0.001**	p = 0.286	p = 0.171	p = 0.207	p = 0.244	p = 0.339	p = 0.055
BDI-II score	**-0.342**	-0.238	**-0.338**	**-0.367**	**-0.403**	**-0.386**	**-0.275**	**-0.385**	**-0.272**	**-0.422**	-0.008	**-0.298**	**-0.486**
	**p = 0.009**	p = 0.071	**p = 0.009**	**p = 0.004**	**p = 0.002**	**p = 0.003**	**p = 0.035**	**p = 0.003**	**p = 0.037**	**p = 0.001**	p = 0.953	**p = 0.022**	**p<0.001**
SSL-I score	0.073	0.125	-0.059	-0.064	-0.073	-0.073	-0.084	-0.066	0.101	-0.110	-0.154	-0.061	-0.082
	p = 0.589	p = 0.354	p = 0.662	p = 0.634	p = 0.590	p = 0.590	p = 0.533	p = 0.627	p = 0.454	p = 0.431	p = 0.251	p = 0.652	p = 0.546
SSL-D score	-0.115	-0.094	-0.140	**-0.275**	**-0.279**	**-0.223**	-0.208	**-0.324**	-0.114	-0.253	0.158	-0.105	**-0.329**
	p = 0.399	p = 0.489	p = 0.302	**p = 0.041**	**p = 0.038**	**p = 0.099**	p = 0.125	**p = 0.015**	p = 0.405	p = 0.071	p = 0.246	p = 0.442	**p = 0.013**

NEI VFQ-25: National Eye Institute Visual Function Questionnaire-25, GH: General Health, GV: General Vision, OP: Ocular Pain, NA: Near Activities, DA: Distance Activities, VSSF: Vision Specific Social Functioning, VSMH: Vision Specific Mental Health, VSRD: Vision Specific Role Difficulties, VSD: Vision Specific Dependency, D: Driving, CV: Color Vision, PV: Peripheral Vision, OCS: Overall Composite Score, VA: Visual Acuity, BDI: Beck Depression Inventory, SSL-I: Social Support List—Interactions, SSL-D: Social Support List–Discrepancies.

* Average of vision-targeted subscale scores, without GH.

Higher LogMAR VA of the worst eye was negatively correlated with general vision and vision specific dependency. Follow-up time was negatively correlated with general vision and vision specific dependency. The BDI-II score was negatively correlated with the OCS and almost all subscales, except general vision and color vision. The SSL-D score was negatively correlated with the OCS and near activities, distance activities and vision specific role difficulties. Positive and negative correlations were seen with higher LogMAR VA of the best eye. Higher LogMAR VA of the best eye was negatively correlated with distance activities, vision specific dependency, peripheral vision and positively correlated with ocular pain. There was no significant correlation between the subscale scores or OCS and the number of uveitis episodes, duration of active uveitis and SSL-I score. The BDI-II score was positively correlated with the SSL-D score (r = 0.486, p<0.001).

## Discussion

In general, the NEI VFQ-25 OCS and subscale scores were relatively high in patients with HLA-B27 associated AU. The mean OCS was 88.9 in the total group. The best score to achieve on each question was 100 and the second best score 75 or 80. This means that most patients scored between the best and second best scores. The OCS is the average of all vision-targeted subscale scores, only excluding general health. The mean general health score was rather low, in comparison to the vision-targeted subscales, namely 47.4. This means that the majority of patients scored their general health between 'fair' (25.0) and 'good' (50.0). Six (10%) of patients had a mild depression.

Patients who were currently being treated for uveitis or ophthalmic complications had lower scores on vision specific social functioning, vision specific mental health, vision specific role difficulties and vision specific dependency. This means that these patients are more worried and frustrated by their eyesight and they need more help because of their eyesight. In our patients, a history of dry eyes seemed to have no effect on the VR-QOL, patients with dry eyes in their history even scored higher on ocular pain (indicating that they experienced less pain or discomfort around or in the eye). In seeming contrast, Li et al. reported that VR-QOL in dry eye patients was significantly impaired. The difference is probably due to the fact that in Li’s study, all patients had dry eyes at the moment of completing the NEI VFQ-25.[13] Whereas in our study, only one patient was being treated for dry eyes at the time of completing the NEI VFQ-25 and 16 patients had a history of dry eyes. So, it seems that current dry eye symptoms and signs may affect VR-QOL outcomes, whereas a history of dry eyes does not. Further, we observed that patients with a longer follow-up time scored worse and patients with a longer remission time scored better on general vision. Longer remission times also seemed beneficial for vision specific mental health.

Subgroup analyses further show that younger patients (<45 years) score better on near activities compared to older patients (≥45 years; Table 2). This is probably due to beginning presbyopia in the older patient group. Having had more than one uveitis episode seems to affect general vision, since patients who experienced only one uveitis episode scored significantly higher on general vision, compared to patients who experienced multiple uveitis episodes. However, by the Spearman’s Rank Correlations test we could not demonstrate an additional effect of the number of episodes. Higher LogMAR VA in either the best or the worst seeing eye was correlated to lower scores on a number of subscales, but not to a lower OCS. The latter was possibly due to the fact that most patients had a relatively good VA in both eyes, and thus differences in VA between eyes and patients were relatively small.

To compare the scores of the NEI VFQ-25 in our patient group to patients with other ocular diseases and healthy persons, and to gain a better insight in the meaning of the scores, we composed Table 5. Based on the vision-targeted subscales, it can be derived that the mean OCS is lowest in patients with age-related macular degeneration.[14] Hirneiss et al. obtained NEI VFQ-25 scores in a normal working population as well as in subpopulations thereof with and without ocular disease. They found that the mean OCS was highest in the working population without ocular disease (91.6), though the difference with the subgroup with ocular disease (mean OCS of 88.8) was small. Our mean OCS (88.9) was comparable to that observed by Hirneiss in the subgroup with ocular disease.[15] Previous studies on non-infectious uveitis found lower mean OCS scores (79.7 and 80.3, respectively) and median OCS score (62.0) than that in our patient group.[4,5,16] A possible explanation is that these studies had included various forms of uveitis, and not just AU. This assumption seems to be confirmed by the relatively low mean OCS (71.0) observed in a study on patients with birdshot chorioretinopathy, which is classified as posterior uveitis.[6] In a previous study, conducted in the same region, we observed that herpetic AU patients are comparable with patients with HLA-B27 associated AU, with regard to the mean OCS (88.1).[7] Based on this comparison, we can conclude that patients with AU associated with either HLA-B27 or herpes, score relatively high on VR-QOL.

**Table 5:** NEI VFQ-25 subscale scores and OCS compared with literature.

			GH	GV	OP	NA	DA	VSSF	VSMH	VSRD	VSD	D	CV	PV	OCS*
Study	Mean age ± SD (yrs)	Group composition						Mean (SD)							
Hoeksema	55 ± 12	HLA-B27 associated	47.4	78.3	73.1	88.0	90.8	97.2	88.7	86.4	97.5	85.3	99.6	94.1	88.9
n = 59		anterior uveitis	(20.8)	(10.8)	(20.3)	(15.2)	(12.3)	(8.7)	(9.2)	(17.4)	(8.9)	(14.2)	(3.3)	(12.6)	(8.8)
Hirneiss 2009^15^	42 ± 9	Normal working population	73.0	78.6	85.4	91.9	91.8	97.9	87.4	92.8	98.4	88.7	97.9	93.3	91.1
n = 619		- Total group	(18.1)	(15.7)	(16.6)	(13.1)	(11.3)	(9.0)	(10.5)	(13.8)	(5.6)	(10.6)	(9.3)	(15.0)	(7.4)
Hirneiss 2009^15^	42 ± 9	Normal working population	79.9	79.0	87.6	92.3	92.1	98.1	87.8	93.4	98.5	88.8	98.0	93.4	91.6
n = 511		- Without ocular disease	(17.4)	(15.9)	(15.1)	(13.0)	(11.4)	(8.2)	(10.0)	(13.3)	(5.5)	(10.6)	(8.7)	(14.6)	(7.1)
Hirneiss 2009^15^	43	Normal working population	68.6	79.1	75.1	90.2	90.6	96.8	85.3	89.7	97.9	88.4	97.3	92.5	88.8
n = 108		- With ocular disease	(20.7)	(15.9)	(19.2)	(13.6)	(10.7)	(12.3)	(12.5)	(15.6)	(5.8)	(10.4)	(11.6)	(16.7)	(8.3)
Hoeksema 2014^7^	58 ± 17	Herpetic anterior uveitis	59.0	76.1	73.3	87.7	92.6	97.1	84.9	84.0	97.7	87.1	97.1	91.2	88.1
n = 36			(19.0)	(9.3)	(22.8)	(16.7)	(12.6)	(8.1)	(14.4)	(21.9)	(7.1)	(16.2)	(10.1)	(20.3)	(10.6)
Qian 2011^4^	41	Noninfectious ocular	60.3	72.8	73.9	79.2	78.8	89.9	70.8	74.2	84.9	77.4	94.9	81.3	79.7
n = 104		inflammatory disease	-	-	-	-	-	-	-	-	-	-	-	-	-
Naik 2013^5^	-	Noninfectious intermediate	59.1	70.3	76.5	78.9	80.5	87.8	71.2	75.3	86.7	81.2	92.8	83.4	80.3
n = 123		and posterior uveitis	-	-	-	-	-	-	-	-	-	-	-	-	-
		(Snellen VA ≥ 0.5)													
Kuiper 2013^6^	59.5 (median)	Birdshot chorioretinopathy	61.6	63.8	75.1	68.6	70.3	84.5	71.2	64.5	84.2	66.8	80.2	67.6	71.0
n = 105			-	-	-	-	-	-	-	-	-	-	-	-	-
Cahill 2005^14^	76.4 ± 5.6	Bilateral severe AMD	-	31.4	81.8	29.4	38.8	58.4	34.1	38.2	42.7	16.1	67.5	66.8	-
n = 70			-	(15.8)	(20.3)	(18.6)	(24.7)	(28.1)	(25.1)	(27.1)	(29.7)	(31.3)	(27.7)	(25.1)	-

NEI VFQ-25: National Eye Institute Visual Function Questionnaire-25. GH: General Health, GV: General Vision, OP: Ocular Pain, NA: Near Activities, DA: Distance Activities, VSSF: Vision Specific Social Functioning, VSMH: Vision Specific Mental Health, VSRD: Vision Specific Role Difficulties, VSD: Vision Specific Dependency, D: Driving, CV: Color Vision, PV: Peripheral Vision, OCS: Overall Composite Score, VA: visual acuity. AMD: age-related macular degeneration.

* Average of vision-targeted subscale scores, without GH.

In contrast, the mean general health score (47.4) in our patient group was relatively low. By comparison, mean general health scores in a normal working population with and without ocular disease were 68.6 and 79.9, respectively.[15] Patients with non-infectious uveitis (general health of 60.3 and 59.1) [4,5] and birdshot chorioretinopathy (general health of 61.6) [6], scored lower than the normal working population and slightly higher than our patient group. Herpetic AU patients from the same referral area as in the present study, also scored higher (59.0).[7] Our results indicate that this may be due to the presence of an HLA-B27 associated systemic disease. In our group, patients with a systemic disease had a mean general health score of 37.5 versus 61.5 in patients without a systemic disease. The latter is comparable to other noninfectious uveitis patients.[4,5] Also, patients with a systemic disease scored lower on VR-QOL. In line with this, studies on the health-related quality of life (Short-Form-36 Health Survey) in ankylosing spondylitis patients, find an influence of systemic disease on reported physical and mental health as well.[17–19] In addition, Kempen et al. suggest that uveitis may have additional health impact over and above its effect on vision, perhaps via symptoms of inflammation unmeasured by visual acuity, side effects of treatment, the impact of associated systemic disease, or a combination thereof.[20]

In our patient group, patients had a mean BDI-ll score of 4.7 (SD ± 5.3; range 0–19) and six (10%) patients had a mild depression at the time of completing the questionnaire. The majority of these, (5/6 (83%)) had ankylosing spondylitis, suggesting that the presence of a systemic disease has a higher influence on this outcome than the presence of AU. Previous studies on HLA-B27 associated diseases, give similar prevalence numbers, since they describe a clinically relevant depression in 12.3% of 171 HLA-B27 associated AU patients and in 14.8% of 55 ankylosing spondylitis patients, respectively.[21,22] Depression may have a negative effect on VR-QOL, since patients with a depression scored significantly lower on the NEI VFQ-25 (Tables 3 and 4). Qian et al. reported that 28/104 (27%) of patients with non-infectious ocular inflammatory disease (including severe posterior and panuveitis patients) screened positive for depression. They observed that these patients scored far lower on the NEI VFQ-25 OCS than non-depressed patients. In their study, inadequate emotional support was highly associated with the development of depression.[4] In our study, depression (BDI-II score) was positively correlated with a deficiency in desired social support (SSL-D score).

The main shortcoming of our study is its modest sample size. Our sample size is considered adequate for overall analyses[23], but it may be too limited for all subgroup analyses, resulting in an underreporting of possibly relevant associations. Also, only 50% of the HLA-B27 associated AU patients participated in the present study. Between participants and non-participants we found that participants were slightly older and had a longer follow-up time, there were no other significant differences, we consider the risk of a selection bias to be small. Furthermore, our patients were seen at a tertiary referral center and therefore this population may not represent the general uveitis population.

In conclusion, patients with HLA-B27 associated AU have a relatively high VR-QOL. However, the presence of a systemic disease is associated with considerably lower VR-QOL scores and general health scores and may be associated with an increased risk of depression. In addition, depression itself is associated with a lower VR-QOL.
